# Risk Factors for Relapse of Human Brucellosis

**DOI:** 10.5539/gjhs.v8n7p77

**Published:** 2015-11-03

**Authors:** Mohammad Reza Hasanjani Roushan, Zahra Moulana, Zeinab Mohseni Afshar, Soheil Ebrahimpour

**Affiliations:** 1 Infectious Diseases and Tropical Medicine ResearchCenter, Babol University of Medical Sciences, Babol, Iran

**Keywords:** brucellosis, relapse, risk factor

## Abstract

**Background & Propose:**

Brucellosis is serious disease around the world, especially in underdeveloped countries. Relapse is major problem in therapy of brucellosis. This study aimed to evaluate risk factors of relapse after treatment in patients.

**Methods:**

It is a descriptive-analytic study from 1990 to 2014, in Ayatolla Rohani hospital in Babol, Iran. We studied 980 patients with brucellosis. The studied community included patients infected with brucellosis and the required information was gathered based on their hospital files. The base for recognizing Malta fever were clinical symptoms and Para-clinical sign congruent with infection like as, titer SAT>1:320 and 2-ME>1:160. Patients with relapse and patients without relapse were placed separately in two groups. The data were statistically compared with Spss 16, by Chi-square and Cox–regression tests.

**Results:**

Based on this study, treatment regimen is a preventive factor (P=0.000). Moreover, Based on some statistical methods, regimens no. 3 and 4 were introduce preventive factors (P=0.001) and (P=0.004). It should also be noted that findings the same statistical model, factors like gender, age, residence, professional contacts, complications and delay in treatment were also analyzed but none of them are considered as preventive factors.

**Conclusion:**

Based our finding, we suggest aminoglycosides (gentamicin or streptomycin with doxycycline) are associated with lower rate of relapse in brucellosis.

## 1. Introduction

Brucellosis is a zoonosis and re-emerging disease which is considered as one of the most important problems of public health in the world. This infection is mainly conveyed to human being through contact with infected animals or consuming animal organs like as liver in a raw or half-baked condition or consuming non-pasteurized dairy like milk, butter, cream and cheese (Dastjerdi, Nobari, & Ramazanpour, 2012; Dean, Crump, Greter, Schelling, & Zinsstag, 2012; Lindahl, Sattorov, Boqvist, & Magnusson, 2015; Poester, Samartino, & Santos, 2013; Young, 1995). More than half a million cases of infection to this disease is reported annually around the world. However, most of the infected countries are located in Middle East, Mediterranean, South America and Indian sub continental (Jia & Joyner, 2015; Pappas, Akritidis, Bosilkovski, & Tsianos, 2005; Supriya, Umapathy, & Ravikumar, 2010). The unsteady appearance of this disease makes its recognition difficult and it can easily be mistaken for other infections. Therefore, confirming the recognition using lab tests based on cultivation, serologic and molecular methods can be quite helpful. In addition, having a thorough record of the patient which includes information like residence, job and eating foods that have highest possibility of conveying the infection is necessary and useful. Treating this disease is done with the goal of eliminating illness symptoms and decreasing the complications like arthritis, spondylitis, miscarriage and stopping the relapse of the infection (del Pozo & Solera, 2012; Smailnejad et al., 2012). To reach this significant goal, various protocols are suggested. Some of them including the one presented by WHO, which is Doxycycline and Rifampin regimen, are compared with many other methods. In all the cases studied, one important criterion which was taken into account in finding a proper cure is the percent of relapse after treatment. According to the definition of experts, relapse is the recurrence of signs and symptoms of illness, with or without the presence of bacteria in blood, after the period of treatment (M. R. H. Roushan, Gangi, & Janmohammadi, 2008). Although many studies refer to the factors that increase the chance of relapse, the best method for controlling the illness and relapse is still unknown. The ability of this micro-organism to live in Macrophages and as a result the inability of the host’s body to access it can justify the relapse and getting more chronic. The measure of relapse occurrence after treatment depends on the type of antibiotics used, their combination and period of treatment. Relapse usually occurs in 5 to 30 percent of patients (Solera, 2010). Differentiating relapse from infection, which is a difficult task to accomplish, is another point of interest to discuss. Clinical signs are usually milder and less exclusive in relapse and biochemical changes happen in a way that are more difficult to recognize. Use of serology and cultivating blood has limited value, though PCR technique seems quite helpful.

According to points just mentioned and importance of relapse in treatment and various factors related to it, studying the factors that are effective in the result of the treatment and relapse shows our goal for this study.

## 2. Methods

### 2.1 Study Population

This data-based descriptive-analytic study was done based on the files of 980 patients in Ayatolla Rohani hospital in Babol, Iran, from 1990 to 2014 who were infected with Malta fever. Entry criteria in this study: active Malta fever; exit criteria: pregnancy, having feverish infections other than Malta fever, incomplete treatment and incomplete follow up.

### 2.2 Research Tools

The studied community included all the patients infected with Malta fever and the required information was gathered based on their hospital files. The base for recognizing Malta fever were clinical symptoms like fever, overnight sweating and Para-clinical sign congruent with infection: titer SAT>1:320 and 2-ME>1:160, all of which were confirmed by the doctor and the doctor considered them as patients and decided to treat their illness. These patients were treated based on regimens 1 to 6. Regimen No. 1 includes Cotrimoxazole and Rifampin, regimen No. 2 includes Doxycycline and Rifampin, regimen No. 3 includes Gentamicin or Streptomycin and Doxycycline, regimen No. 4 includes Gentamicin and Doxycycline, regimen No. 5 includes Cotrimoxazole and Doxycycline and regimen No. 6 includes other medicines. Presence or absence of relapse in each patient was confirmed by the doctor considering clinical and para-clinical factors, and recurrence of clinical symptoms in 6 months, with or without bacteria, along with increase in titer wright and 2ME which was decreased previously, was considered as relapse (M. H. Roushan et al., 2010). All this data were registered in special forms designed for this study.

### 2.3 Data Analysis

The extracted information was analyzed with Spss software. Chi-square and Cox–regression tests were used. The difference between data was considered statistically significant at p-value lower than 0.05.

## 3. Results

Out of 980 patients studied, 570 (58.1%) were men and 410 (41.9%) were women. Mean age was 33.5±16 range was 1to 90years. About the place of residence, 561 (57.2%) resided in the city and 419 (42.8%) resided in the country. Moreover, 427 (43.5%) were in contact with animals. Based on this study, treatment regimen is a preventive factor (an element designed to stop relapse or ill health from occurring) (P=0.000) (Table 1). Based on the statistical method of Cox regression, regimens No. 3 and 4 were introduce preventive factors (P=0.001) and (P=0.004) (Table 2). It should also be noted that based on the same statistical model, factors like gender, age, residence, professional contacts, complications and delay in treatment were also analyzed but none of them are considered as preventive factors (Table 2). Among the various factor like demographic characteristics, clinical symptoms, some para-clinical findings and treatment regimens which were studied, of the 570 men, 57 (10%) had a relapse. Of the people older than 40 (665 patients), 63 (9.5%) had a relapse. Of the 561 urban patients, 66 (11.8%) had a relapse. Those who had delay in treatment in less than 2 month or756 patients, 79 (10.4%) had a relapse. Considering clinical symptoms among feverish patients which were 574, 52 (9.5%) and considering overnight sweating in 595 cases, 54 (9.1%) patients had a relapse. Among patients without complication or 720 patients, 74 (10.3%) patients showed a relapse. Also in cases with wright test less than 1:640 namely, 503 patients, 48 case (9.6%) had a relapse. Moreover, findings in Table 1 were compared with Chi-square and notes in Table 2 were analyzed with Cox –regression in SPSS.

**Table 1:** Comparison of 980 patients with brucellosis who have or have not endured a relapse

		Number	(relapse)	Non (relapse)	P-value
Total		980	10.51%(103)	89.49%(877)	

Male	Sex	570	10%(57)	90%(513)	.598
	Female	410	11.2%(46)	88.8%(364)	
Age	<40	665	9.5%(63)	90.5%(602)	.147
	>40	315	12.7%(40)	87.3%(275)	
Residency	Urban	561	11.8%(66)	88.2%(495)	.142
	Rural	419	8.8%(37)	91.2%(382)	
Occupational exposure		427	9.4%(40)	90.6%(387)	.345
Delayed treatment	<2month	756	10.4%(79)	89.6%(677)	.8
	2month -1year	185	10.3%(19)	89.7%(166)	
	>1year	39	12.8%(5)	87.2%(34)	
Fever		574	9.5%(52)	90.5%(493)	.295
Chills		227	9.3%(21)	90.7%(206)	.538
Sweating		595	9.1%(54)	90.9%(541)	.071
Weakness		149	7.4%(11)	92.6%(138)	.194
Fatigue		123	8.1%(10)	91.9%(113)	.433
Anorexia		46	8.7%(4)	91.3%(42)	1
Nausea and vomiting		12	8.3%(1)	91.7%(11)	1
Diarrhea		5	20%(1)	80%(4)	.427
Myalgia		345	9.9%(34)	90.1%(311)	.664
Arthralgia		511	11.4%(58)	88.6%(453)	.405
Headache		90	6.7%(6)	93.3%(84)	.278
Abdominal pain		15	6.7%(1)	93.3%(14)	1
Back Pain		275	13.5%(37)	86.5%(238)	.64
sacralgia		36	13.9%(5)	86.1%(31)	.417
Sacroiliitis		75	9.3%(7)	90.7%(68)	.846
Arthritis		144	11.1%(16)	88.9%(128)	.77
Epididymo-orchitis		46	15.2%(7)	84.8%(39)	.319
Splenomegaly		55	5.5%(3)	94.5%(52)	.262
Skin lesion		10	10%(1)	90%(9)	1
Abortion		4	0%	100%(4)	1
Weight loss		73	6.8%(5)	93.2%(68)	.425
Lymphadenopathy		23	17.4%(4)	82.6%(19)	.291
Complications	Yes	260	11.2%(29)	88.8%(231)	.724
	No	720	10.3%(74)	89.7%(646)	
Positive blood culture		5	0%	100%(5)	1
Wright test	< 1:640	503	9.6%(48)	90.4%(455)	.403
	> 1:640	477	11.3%(54)	88.7%(423)	
Treatment regimen	No.1	181	14.4%(26)	85.6%(155)	0
	No.2	78	17.9%(14)	82.1%(64)	
	No.3	343	5.5%(19)	94.5%(324)	
	No.4	129	.8%(1)	99.2%(128)	
	No.5	185	19.5%(36)	80.5%(149)	
	No.6	64	10.9%(7)	89.1%(57)	

**Table 2:** Comparison preventive factors of relapse in patients with brucellosis

		HR (CI:95%)	P-value
Sex		.957(.643-1.424)	0.829
Age		1.353(.899-2.035)	0.147
Rural		.871(.580-1.307)	0.505
Occupational exposure		1.001(.644-1.508)	0.998
Complication		1.091(.702-1.694)	0.699
Delayed treatment	<2month	1.149(.688-1.918)	0.596
	2month-1year	.982(.393-2.455)	0.968
Treatment regimen	No.1	1	-
	No.2	1.219(.629-2.364)	0.557
	No.3	.359(.196-.657)	0.001
	No.4	.051(.007-.379)	0.004
	No.5	1.375(.825-2.292)	0.222
	No.6	.718(.310-1.664)	0.439

* HR: Hazard Ratio.

## 4. Discussion

As mentioned, Malta fever is a major problem for public health in some countries including Iran. Eliminating this bacterium is the main method for treating this disease for clinicians. In order to do this, defensive mechanisms of body, especially cellular security, act along with innate safety, although because this micro-organism is Intracellular, security system and treatments are helpful and chronic infection or relapse happens. In the present study, various factors of danger including demographic characteristics, clinical symptoms, some para-clinical findings and treatment regimens were studied for Malta fever relapse, among which treatment regimen was introduced as a preventive factor. In other words, regimens no. 3 and 4 prevented relapse in this community. Using Gentamicin and Doxycycline or Streptomycin with Doxycycline had acceptable results for us in this study. Nowadays, WHO suggests a combination of Gentamicin and Doxycycline as an acceptable regimen for treating community (Pappas, Akritidis, & Tsianos, 2005). In a study by Solera et al. it was shown that using Doxycycline for 45 days and Gentamicin for one week severely decreases relapse (Solera et al., 2004). In another study by Lubani et al. which was done on children it was shown that using Doxycycline for 3,5,8 weeks with using Gentamicin for 5 days had no relapse (Lubani et al., 1989). WHO has suggested using Streptomycin along with Doxycycline. Although some studies including ours confirm this, some believe that it has high measure of relapse. This regimen is effective based on what was said anyways (Ariza et al., 2007). In similar studies, using a combination of Streptomycin and Doxycycline is considered a proper regimen with low measure of relapse. Erosy et al. mentions this in their study (Ersoy, Sonmez, Tevfik, & But, 2005). In another study by Alp et al. similar result were presented (Alp et al., 2006). However, in studies like those done by Hasanjani roushan et al. Doxycycline was used for 45 days, along with using Streptomycin for 14 days or Gentamicin for one week and it was shown in the end that combination of Doxycycline and Gentamicin has the same treatment effect as using Doxycycline and Streptomycin and both regiments are effective for treating the infection (M. R. H. Roushan, Mohraz, Hajiahmadi, Ramzani, & Valayati, 2006). In another study by Hasanjani roushan et al. which compared the effectiveness of using Gentamicin for 5 days and Doxycyclinefor 8 weeks versus using Streptomycin for 2 weeks with Doxycycline for 45 days, the effectiveness of these regimens and their equality was also indicated (M. H. Roushan et al., 2010). It should be taken into account that many studies using other treatment regimens, like the study done by Mantur et al. were done and achieved acceptable results and it was seen that in Gentamicin, Doxycycline and Rifampin regimens, no relapse was observed (Mantur et al., 2004), Or in a study by Hashemi et al. the effectiveness of Doxycycline with Rifampin and Ofloxacin with Rifampin in a lower level than Doxycycline and Streptomycin is indicated (Hashemi et al., 2012).

## 5. Conclusion

Since the main and exact reason for Malta fever is not recognized yet, and considering its importance in treatment, studying the reasons and causes of this disease along with trying to find clinical parameters and lab methods related to relapse can be quite helpful. Therefore, further studies are required in order to reach this goal.
